# Modeling Symmetric Macromolecular Structures in Rosetta3

**DOI:** 10.1371/journal.pone.0020450

**Published:** 2011-06-22

**Authors:** Frank DiMaio, Andrew Leaver-Fay, Phil Bradley, David Baker, Ingemar André

**Affiliations:** 1 Department of Biochemistry, University of Washington, Seattle, Washington, United States of America; 2 Department of Biochemistry, University of North Carolina, Chapel Hill, North Carolina, United States of America; 3 Fred Hutchinson Cancer Research Center, Seattle, Washington, United States of America; 4 Department of Biochemistry and Structural Biology, Centre for Molecular Protein Science, Chemical Centre, Lund University, Lund, Sweden; University of South Florida College of Medicine, United States of America

## Abstract

Symmetric protein assemblies play important roles in many biochemical processes. However, the large size of such systems is challenging for traditional structure modeling methods. This paper describes the implementation of a general framework for modeling arbitrary symmetric systems in Rosetta3. We describe the various types of symmetries relevant to the study of protein structure that may be modeled using Rosetta's symmetric framework. We then describe how this symmetric framework is efficiently implemented within Rosetta, which restricts the conformational search space by sampling only symmetric degrees of freedom, and explicitly simulates only a subset of the interacting monomers. Finally, we describe structure prediction and design applications that utilize the Rosetta3 symmetric modeling capabilities, and provide a guide to running simulations on symmetric systems.

## Introduction

Homomeric protein assemblies are ubiquitous in nature, playing many key roles in biochemical processes. These assemblies are built up by the repetition of a single structural unit, the most common example being homodimers with two protein subunits. Homomeric assemblies often play a morphological role by forming channels, containers and molecular rulers. Almost all homomeric assemblies have a symmetrical arrangement of their subunits in three-dimensional space. Symmetry is a central concept in understanding the structural organization of many protein complexes and is fundamental to the field of crystallography.

Due to the biological importance of symmetrical protein assemblies, the need arises to structurally model symmetrical protein systems. Symmetry imposes fundamental constraints on the organization of these protein assemblies, which enables the computational treatment of very large systems. In this work, we describe a general framework for modeling arbitrary complex symmetries in Rosetta3. First, we give a short background to the different types of symmetries that are relevant to the study of protein structures and crystallography. Then we describe how this symmetry machinery is implemented within Rosetta: we restrict the conformational search space by sampling only symmetric degrees of freedom, and systems are limited by only explicitly simulating a subset of the interacting monomers. Optimizations that allow efficient scoring and minimization of symmetric systems are described. We proceed by providing a guide to running symmetric simulations with Rosetta. We describe several tools by which one may how one may define a symmetric system, and how several Rosetta protocols may be run in the context of symmetric partners. These protocols include docking, ab initio structure prediction, comparative modeling, and protein design. Finally, we compare the performance of the symmetry machinery in Rosetta3 with the implementation in Rosetta2 [1] and provide estimates of how running time scales with number of subunits in the symmetrical system.

### Background

**Figure 1:**
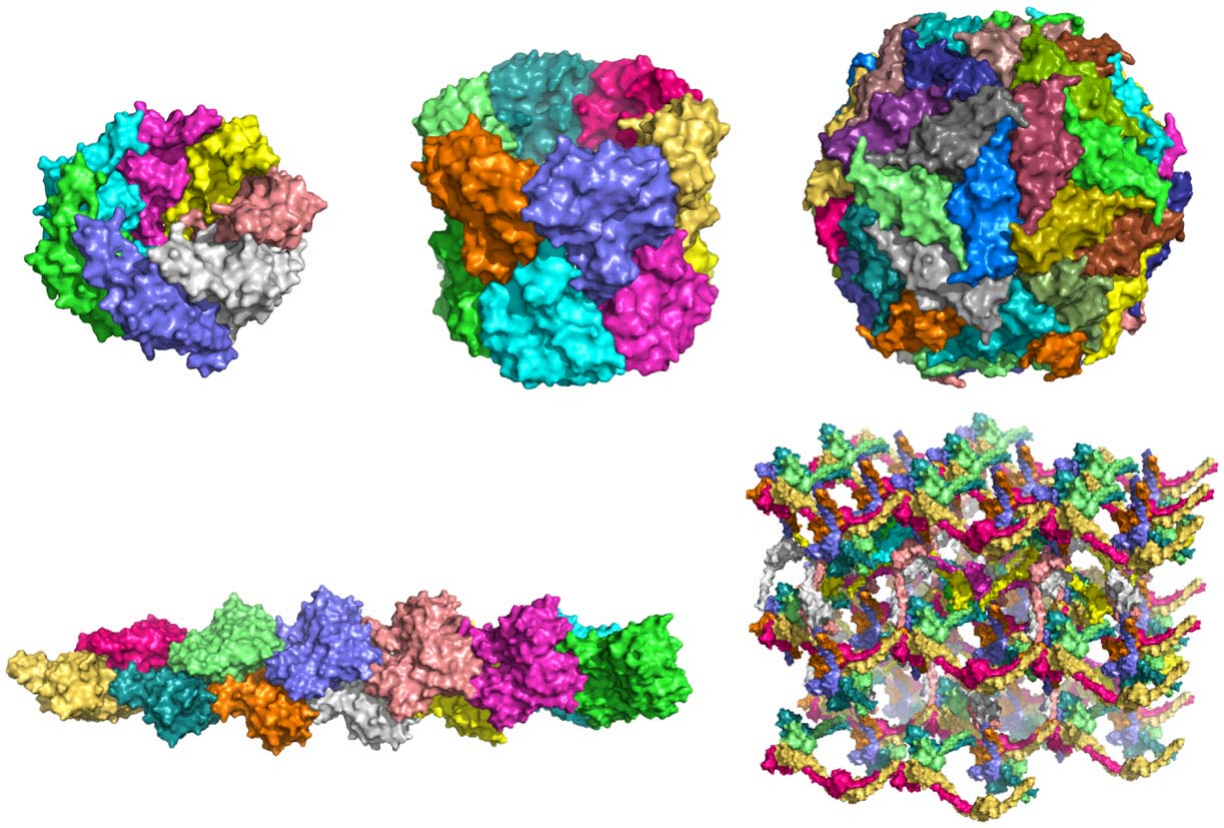
An overview of various types of symmetry present in the PDB, that can be modeled using Rosetta's symmetric modeling framework. **(top)** Point symmetry groups C*_n_* (PDB id 1tg6), D*_n_* (PDB id 1znn), and icosahedral (PDB id 1stm). **(bottom)** Lattice groups showing helical (PDB id 3g37) and crystal (PDB id 3m9b) repeats.

Regular symmetries include point, helical and crystal symmetries. Figure 1 illustrates the various symmetry groups, as well as illustrating the symmetric degrees of freedom needed to define these systems.

#### Point Symmetry

There are five basic types of point symmetry, denoted by Schöenflies symbols C, D, T, O, and I. The most common type of symmetry is cyclic, or C*_n_*, symmetry. Here, a symmetric complex is comprised of a set of subunits arranged in a ring about a single rotation axis (a dimer, or C_2_, is a special case of this symmetry). Complexes with high-order cyclic symmetry are used as ring structures in pores and in chambers. Dihedral, or D*_n_*, symmetry, is also commonly observed in natural biological assemblies. In this symmetry, two C*_n_* symmetry groups form a dimer, with symmetry axis perpendicular to that of the C*_n_* group. As an example, a clathrin cage (pdb id 1xi4) exhibits D_6_ symmetry; D symmetry provides additional interface variety that leads to more stability as well as improved allosteric control. The higher-order symmetries T, O, and I consist of three-fold symmetry groups at the vertices of a tetrahedron, octahedron, and icosahedron, respectively. Icosahedral symmetry is very commonly observed in viral structures, as it produces roughly spherical assemblies, suitable for storage and transport. Tetrahedral and octahedral symmetries are less common, but have been observed in ferretin structures.

#### Helical Symmetry

Helical symmetries are produced by rotation and translation along a single symmetry axis and have been observed in microtubules, flagella and actin filaments. As well, amyloid fibers displaying helical symmetry are associated with a number of diseases, such as Creutzfeldt-Jacob's disease and Alzheimer's disease. In simple helical symmetries, only three parameters (aside from the orientation of a reference subunit) are required to uniquely define the symmetric system: an angle of rotation between subunits, a translation (or “rise”), *Z*, along the helical axis per subunit, and a distance *X* from the helical axis to a reference point on each subunit. In more complex cases each subunit is replaced with a C*_n_* point group (polar helical symmetry) or a D*_n_* point group (nonpolar helical symmetry).

#### Wallpaper and Crystal Symmetry

Wallpaper and crystal symmetries occur when a subunit forms a repeating two-dimensional or three-dimensional pattern. There are 17 possible two-dimensional repeats, and – ignoring cases impossible by protein's chirality – 65 possible three-dimensional repeats. These are referred to as *spacegroups*. For three-dimensional spacegroups, in addition to the spacegroup itself, anywhere from six to nine parameters are needed to describe the symmetric system: one to six of which describe the size and shape of the repeating unit, and three to six which describe the rigid-body orientation of a reference subunit. For two-dimensional spacegroups, one to four parameters describes the repeating unit, and one to three describes the rigid-body orientation of a reference subunit. This formation of three-dimensional crystal repeats is key to solving structures through X-ray crystallography.

The presence of symmetry leads to large reduction in the number of parameters required to describe the relative orientation of protein subunits in coordinate space. For an asymmetric system the number of degrees-of-freedom required to specify an oligomer is 6×(number of subunits -1), while a symmetrical system can typically be described with 3 to 6 degrees-of-freedom.

## Methods

All modeling tasks in Rosetta consist of two general components: *conformational sampling* and *energy evaluation*. Both of these components may take advantage of symmetry. Thus, the implementation of the symmetry machinery in Rosetta has been driven by two guiding principles: first, to reduce the conformational search space by only sampling conformations consistent with the given symmetry, and second, to perform only the minimal number of pairwise energy evaluations necessary to capture the total energy of the symmetric system. To achieve this, a number of core elements of the Rosetta program have been adapted: *(i)* kinematics, which describes how changes in internal degrees of freedom propagate in the system, *(ii)* energy evaluation, *(iii)* discrete side-chain optimization, and *(iv)* energy minimization.

### Kinematics for symmetric systems

The conformation of a macromolecule in Rosetta is represented by a tree-like structure, with either atom-level (*atom trees* in Rosetta) or residue-level (*fold trees*) connections. For simplicity, we will only consider the fold tree representation for this document. A fold tree is a directed acyclic connected graph composed of peptide segments together with long-range connections [2], [3]. Each residue is a vertex in this graph, connected with peptide edges to preceding and following residues. At chain breaks, such as those in a multi-protein assembly, or artificially introduced during modeling, long-range connections (*jump edges* or *jumps*) specify the relative rigid-body orientation of non-covalently attached peptide segments. The fold tree is defined by selecting a root vertex; the conformation of downstream residues is calculated, extending from the root residue, by traversing the edges and jumps in the fold tree.

The conformational degrees of freedom (dofs) of a molecular system are the torsion angles of the backbone and side-chains along with the rigid-body transformations between peptide segments. Maintaining perfect symmetry with regards to the internal structure of protein subunits is straightforward: when a torsion angle is set in one subunit, it is simultaneously set in all other subunits. For implementation purposes, we describe the internal degrees of freedom with respect to a *master* and one or more *slave* subunits. Only in the master subunit may torsion angles (or other internal degrees of freedom) be set, and when torsions are set in the master subunit they are immediately propagated to the corresponding degree of freedom in the slave subunit. The master subunit must be carefully selected, as it plays a key role in energy calculation: it must be surrounded by all the interaction partners that need to be present in order to calculate the total energy of the symmetric system (see more on this in the section on energy calculations).

Maintaining rigid body symmetry between subunits is more challenging. The representation effects both energy evaluation and minimization; this representation must be general enough to model arbitrary complex symmetries. With Rosetta, we have opted for a system in which the rigid body configuration of each subunit is controlled by its own reference frame. These reference frames are related to one another by symmetry operations defined by the symmetry group. Analogous to how the identity of the internal structure between subunits are maintained, a change of coordinates of one subunit relative to its reference frame is replicated to all other subunits/coordinate systems, enforcing rigid body symmetry. The position of a subunit relative to its reference frame is controlled by jumps. These jumps are described by 6 variables, three rotational and three translational, that describe the rigid-body transformation between the start and end coordinates of the jump. These reference frames are implemented in Rosetta by introducing non-amino acid pseudo residues, called *virtual residues*, which can be incorporated into the fold tree in the same way as an amino-acid residue. Using these virtual residues, the rigid body positions of subunits are defined by jumps from each virtual residue to an anchor residue in the protein. When a jump is set to the protein from a master virtual residue the jump is replicated to the slave virtual residues, which apply the jump to their attached anchor residue. This replicates rigid body changes in the master to all slave subunits.

To maintain the overall symmetry of the system, for many symmetry groups only a subset of translations/rotations are allowed to move. The reference frames are set up such that – if rigid-body movement is restricted – the allowed direction of movement coincides with one of the principle axes of the virtual residue (e.g., rotation around the *x*, *y* or *z* axis and translation along *x*, *y* or *z*). Generally, symmetry restricts the standard 6 dofs into a smaller allowed set; for example, a C_2_ symmetric system may be set up such that only translation along *x* or rotation about any axis is permitted.

In many applications (such as *ab initio* structure prediction) the absolute coordinates of the symmetric system are irrelevant. However, in other cases, it may be necessary to maintain a global reference frame for the entire system. For example, this arises when modeling membrane proteins (where we care about the protein's position relative to a membrane plane) and may also arise in the context of additional experimental data, such as electron density or residual dipolar coupling (RDC) data. To handle these cases, in addition to the “virtual” reference frames that control the symmetry in the system, an additional virtual residue is added as a root reference frame, which controls the global coordinates of the symmetric system.

**Figure 2:**
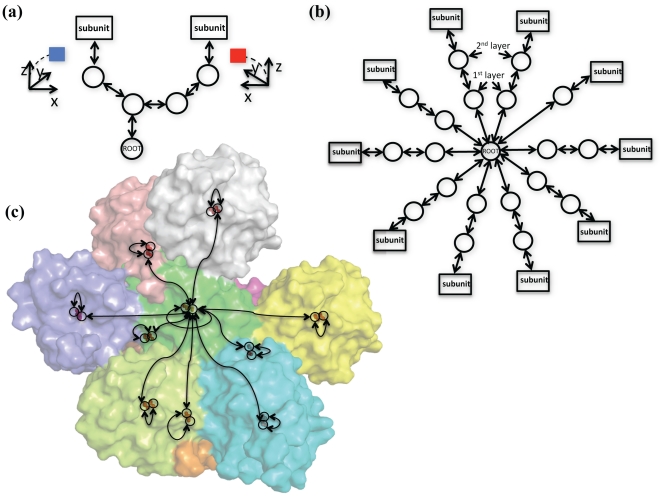
Illustrations of the setup of virtual frames responsible for maintaining rigid body symmetry. Circles represent virtual residues and arrows beween indicate a jump. **(a**) The standard setup of virtual residues to generate a C_2_ symmetric protein complex (see Figure 8a to see a symmetry definition file that generates this setup). The virtual labeled ROOT is the root of the FoldTree and controls the absolute coordinate system. The terminal vertices are connected by a jump to an anchor residue in the protein subunits and the reference frames encoded by these two virtual residues are related to each other by a twofold rotation around an axis. **(b)** Setup of virtual residues to encode for the *I* 2 2 2 spacegroup symmetry. The virtual labeled ROOT is the root of the FoldTree. Jumps from the root virtual to the first layer of virtual residues control the overall placement of the 10 subunits in 3D space. These jumps can be used to orient the asymmetric unit into experimental electron density. The jumps from the first layer to the second can be used to move the subunits in the unit cell while maintaining the space group symmetry. The jump from the second layer to the subunits can be used to rotate the subunits around their center of mass. **(c)** Placement of virtual residues in three-dimensional space for the *I* 2 2 2 spacegroup (taken from pdb id 1x6j). The crystal is represented by 10 subunits. Virtual residues are shown as rings (and red spheres) and arrows illustrate jumps.

The setup of virtual residues (which act as reference frames) is described in a tree-like hierarchy, like that shown in Figure 2(a). In this case, the virtual residues at the bottom of the tree are connected by a jump to each subunit; this jump controls the rotation of each subunit. Another jump connects these virtual residues to another set of virtual residues, which controls the separation between subunits. Finally, if a global reference frame is required, this intermediate layer would then be connected by jumps to a single root virtual residue. In problems where the absolute coordinate system is irrelevant, this virtual residue may be excluded. Figure 2(b-c) show an alternate symmetric system, where the virtual residues are set up for modeling a structure with crystallographic symmetry. By assuming fixed unit cell size, we don't have to worry about moving the jumps between virtual residues, greatly simplifying the setup of our system.

### Efficient evaluation of symmetric structures with Rosetta's full-atom energy function

The framework outlined in the previous section describes how kinematics are enforced and propagated in symmetric poses. In this section, we briefly introduce how the energy of structures are evaluated in Rosetta. We describe several modifications to energy evaluation that allow for increased efficiency when evaluating structures known to be symmetric. Since scoring takes the majority of time in most Rosetta full-atom protocols, these enhancements result in a significant increase of speed in almost all modeling and design protocols.

Rosetta's fullatom energy function is comprised of a linear combination of terms. For implementation purposes, these energy terms are divided into four separate classes: one-body energy terms, distant-dependent two-body energy terms, distance-independent two-body energy terms, and whole-structure (or “many-body”) energy terms [4].

**Figure 3:**
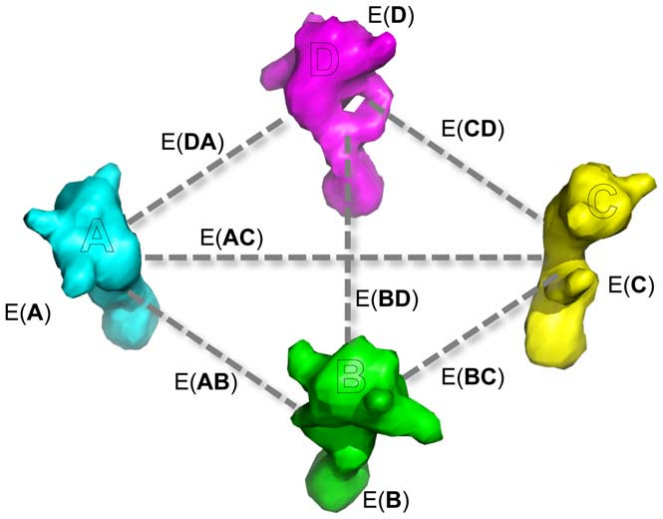
An example illustrating energy calculation for a C_4_ symmetric system. Of the six interfaces in the symmetric complex, only two of these are unique: the energy of interface AB is identical to that of BC, CD, and DA; the energy of interface AC is identical to that of BD. The energies internal to one subunit are identical to those in each of the symmetric copies. Thus, to compute the energy of the entire system, we only need consider the internal energy of A and the energy of interfaces AB and AC.

When scoring symmetric structures, we quickly notice that a majority of these interactions are duplicated multiple times throughout the complex. For example, if we consider the C_4_ system of Figure 3, with subunits A-B-C-D, we see that the internal interactions of subunit A are repeated throughout the other three subunits. The interactions between A and B are repeated four times throughout the system (B-C, C-D, and D-A are identical); the interactions between A and C appear twice (B-D is identical).

Thus, ignoring whole-structure energies, we see that in order to evaluate the energy of a symmetric complex, we only need to consider the energy of one subunit (the master subunit), plus the interactions that subunit makes with each of the other subunits. Revisiting the C_4_ system in Figure 3, the energy of the complex is given as:
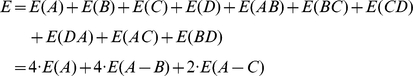



Here E(*XY*) refers to the interaction energy between residues in subunit X and subunit Y.

**Figure 4:**
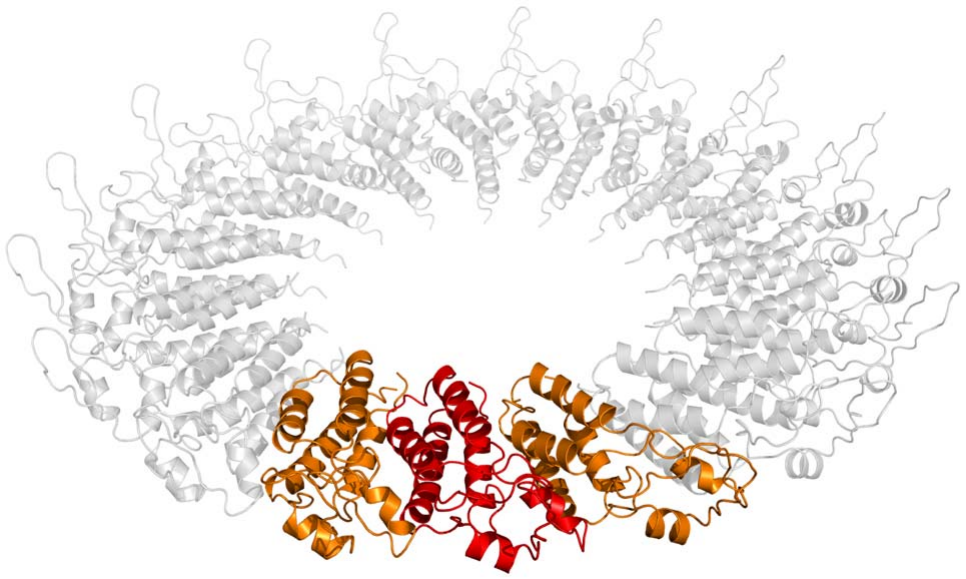
An illustration showing how we may compute energies of large symmetric complexes with only a few subunits explicitly modeled. In the C_17_ system shown here (PDB id 3kml), if we assume interactions at a distance of more than 10 Å contribute a negligible amount of energy, then we only need to model the three colored subunits in Rosetta. The entire system's energy (and gradients) may be described in terms of the energy of the master subunit (red) and the interactions between the master and the adjacent slave subunits (orange).

Furthermore, if we assume that there is a maximum interaction distance of any two-body energy function, then we only need to explicitly model subunits whose residues will possibly approach to within this maximum interaction distance during simulation. For example, when modeling a large ring, like the C_17_ structure shown in Figure 4 (PDB id 3kml) we only need to explicitly model 3 subunits in order to accurately compute the energy of the entire system, assuming that interactions over distances greater than 10 Å contribute a negligible amount to the total system's energy. For an icosahedral virus capsid, generally only 6 of the 60 subunits need to be explicitly modeled to accurately recapitulate the total capsid energy. This allows for efficient modeling of extremely large symmetric assemblies.

Note that if we are calculating agreement with experimental data that are dependent on the conformation of the entire complex, such as residual dipolar coupling (RDC) data or small-angle X-ray scattering (SAXS) data, then all subunits must be explicitly included in order to correctly evaluate these whole-structure energies.

#### Building a restricted energy graph

One time-consuming step in scoring a structure is computing the energy graph for the distant-dependent two body energies. Here, we must compute all pairs of residues containing atoms within some cutoff distance of one another. For asymmetric structures, Rosetta represents this cloud of atoms with an octree. Using an octree, the energy graph of a protein with N residues is computed in two steps: first the octree is constructed from the “atom cloud,” then, for each residue in the protein, the nearby residues are found. With a symmetric structure, we only need to consider edges in this energy graph with at least one vertex in the “master” subunit. Assuming we are explicitly modeling S subunits, then we only need to query the octree N/S times instead of N times (the time spent constructing the octree is the same). This speedup is particularly noticeable in cases where experimental data requires that a large number of subunits be explicitly modeled.

#### Implementation of symmetric energy evaluation

The total energy of a symmetric system is given in terms of interface energies as a line in the symmetry definition file, for example, the symmetry definition file for the C4 system in Figure 3 would contain:
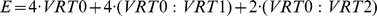



Here, *VRT0* is the virtual residue anchoring the master subunit (‘A’ in Figure 3), and *VRT1* and *VRT2* anchor neighboring subunits (‘B’ and ‘C’ in Figure 3, respectively). The section on symmetry definition files describes this syntax in more detail.

When scoring a symmetric structure, Rosetta attaches a weight to each interaction edge. For one-body energies and two-body energies within the master subunit, this weight is simply the weight on master subunit (‘4’ in the example above). For two body energies between the master and some other subunit, the weight is the corresponding weight from the symmetry definition file: in this case, ‘4’ for interactions with the slave subunit controlled by virtual residue *VRT1*, and ‘2’ for interactions with the slave subunit controlled by virtual residue *VRT0*.

#### Whole structure energies

Whole-structure energies are slightly trickier to handle within the symmetric framework. In many cases, it is not clear whether a more suitable interpretation is to compute the energy over one subunit and scale this energy by the number of subunits, or to compute these energies over the whole symmetric complex and leave it unscaled. We have opted for the latter, with the justification that scoring a complex with point symmetry should give the same results using symmetric scoring as asymmetric scoring. However, with lattice symmetry, or cases where only some subset of the complete system is explicitly modeled, these whole-structure energies may not make much sense. Therefore, this behavior may be modified for particular score functions by making the appropriate corrections in SymmetricScoreFunction::correct_finalize_score().

### Symmetric Packing

Rosetta's sidechain optimization module, the packer, can also take advantage of the same efficiencies that make scoring rapid. In asymmetric sidechain optimization, the packer builds a discrete set of rotamers [5] at n positions on the structure, and attempts to solve
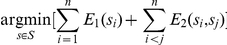
where *S*  =  ∏*S_i_* is the Cartesian product of the individual residue rotamer state spaces; *s* is a rotamer assignment with *s_i_* ∈ *S_i_* representing the rotamer assigned to residue *i*; 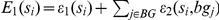 represents the sum of the rotamer internal energies (*ε*_1_) and its pairwise interaction energies (*ε*_2_) with the background (*bg*) residues; and, the two-body energy, *E*_2_(*s_i_*,*s_j_*), is the rotamer-pair energy *ε*_2_(*s_i_*,*s_j_*) between rotamers *s_i_* and *s_j_*. This problem is NP-Complete [6], so Rosetta uses a stochastic algorithm [7]. The sidechain placement problem may be readily abstracted to a state assignment problem where states must be assigned to nodes in a graph [8]. In this abstraction, node energies replace *E*_1_ and edge energies replace *E*_2_. For speed, Rosetta precalculates and stores the node and edge energies in a sparse interaction graph for rapid retrieval.

The state assignment problem is also a fine model for the symmetric packing task, where, assigning rotamer 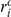 to a residue *i* in the master subunit, *c*, must correspond to the assignment of the symmetrically similar rotamers 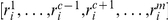 to the other m subunits. In this case, a single state *s_i_* corresponds to a collection of rotamers 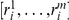, implying that the node energy for state *s_i_* is given by
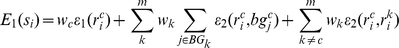
where *w_c_* is the weight given for the intra-subunit interactions, and *w_k_* is the weight between the master subunit and subunit *k*. Of course, if *w_k_*  =  0, then the energies between 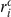 and the background residues on subunit *k* need not be evaluated. The third term in this equation accounts for the two-body energies between a rotamer and its symmetric copies.

Similarly, the edge energy for states *s_i_* and *s_j_* is given by
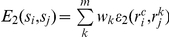



With these equations for calculating the node and edge energies, the same interaction-graph data structure and the same discrete-optimization algorithm used to solve the asymmetric sidechain placement problem may be used.

### Efficient minimization of symmetric systems

In this section, we describe the basic framework we use when minimizing symmetric systems. We discuss a few implementation issues, and describe two cases that require special treatment: lattice symmetries, like that of helical symmetry or 2D or 3D crystal tilings, and asymmetric whole-structure energies.

**Figure 5:**
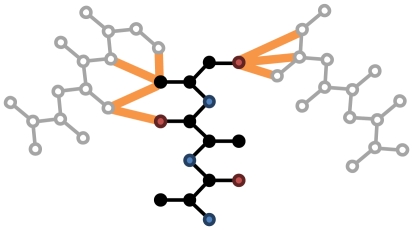
An illustration of our minimization strategy. Since every subunit is in the same symmetric context, we only need to consider gradients with respect to the master subunit. Thus, when computing derivatives with respect to the motion of *all* symmetric copies of an atom, we only consider *the master subunit* (shown in color) and its interface with all neighboring subunits (shown in grey). In the local coordinate system of the slave subunits, the corresponding gradients are identical. In the illustration below, the orange lines indicate the interface edges along which inter-chain derivatives are computed.

As with kinematics and scoring, minimization of symmetric complexes is done with respect to a master subunit. For each backbone torsion and rigid-body degree of freedom in the master subunit, we compute the derivative of Rosetta's all-atom energy with respect to the corresponding degree of freedom. Figure 5 illustrates our strategy for symmetric minimization. When minimizing systems with point symmetry within Rosetta (lattice symmetries are slightly more complicated; see below), as with scoring, we only consider interactions with the master subunit. Unlike scoring, derivatives with respect to all interactions – not just the unique interactions – are computed; these interactions are all weighted equally. The reason for this difference is straightforward: using the C_4_ case in Figure 3 as an example, while two-body energies across the interface between A and B are identical to those of D and A, the directions of the gradients are different.

Formally, we compute the partial derivative of the energy E of a system with point symmetry (where *x_i_* is a degree of freedom, **R_k_** is the transformation from subunit k to the master subunit, 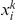 is a symmetric copy of d.o.f. *x_i_*, and 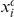 is the master subunit's copy of this d.o.f.):
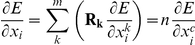



As with asymmetric minimization, the formulation of Abe et al. [9] is used to efficiently convert Cartesian derivatives into torsion-space derivatives. Rigid-body rotation is handled by treating the rotation as one or three “pseudo-torsions”: three in cases where the orientation is free to move, and one in cases where the orientation may only spin about a particular axis to maintain the overall symmetry of the system.

#### Minimization with lattice symmetry

**Figure 6:**
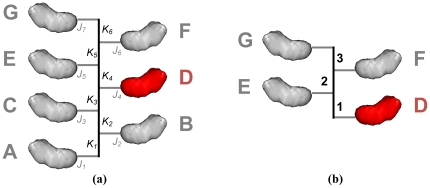
When modeling helical symmetry, special treatment must be given when minimizing the degree of freedom corresponding to the helical rise. **(a)** A helical system in Rosetta is configured such that the *J_i_* jumps are all cloned, as are the *K_i_* jumps (the master subunit, D, is shown in red). When minimizing *with respect to the K_i_ jumps* we only need to consider derivatives with respect to one side of the symmetric interface; gradients with respect to these jumps must be weighted when an interface passes through multiple copies of this jump. **(b)** The solution is to store a weight with each cloned jump. These weights correspond to the scaling of downstream movement with respect to movement of the cloned *K_i_* jump. For example, gradients between D and F pass through two copies of the cloned *K_i_* jump; movement of the *K_i_* jump by a vector *x* causes the vector between D and F to move by 2*x*.

When minimizing with respect to lattice symmetry, additional complication arises when minimizing the degree of freedom corresponding to the rise between subunits. One issue that arises is that gradients along the rise of the helix only should be computed in one direction only. For example, consider a helix containing seven subunits, *A*-*G*, as in Figure 6. If we consider subunit *D* as the master subunit, then the derivatives with respect to the helical rise across the interface of *D* and *C* will be canceled by the derivatives across the interface of *D* and *E* (as they will all be the same magnitude but in the opposite direction).

A second issue has to do with gradients across an interface that spans multiple copies of the helical rise “jump.” Consider the interactions of subunits *D* and *E* with respect to the degree of freedom representing the rise of the helix (that is, the vertical distance between subunits. Increasing the rise by 1 Å has a corresponding increase of 1 Å in the distance between subunits *D* and *E*. However, if we instead consider subunits *D* and *F*, we see that increasing the helical rise by 1 Å causes the distance between subunits to increase by 2 Å, because the interaction passes through two “copies” of the jump corresponding to the helical rise. Only one of these jumps corresponds to a degree of freedom in the system, so, naively, Rosetta treats these two cases identically.

To account for this, each cloned jump has a weight associated with it. This weight specifies a scaling factor that is applied to derivatives coming into the jump, before they get remapped to the master jump. Thus, the derivatives computed in the interface between *D* and *F*, as they propagate through the jump marked *K*_5_, are scaled by a factor of 2 (corresponding to the number of copies of the cloned jump between *D* and *F*). The gradients computed between the interface of *D* and *F* are then equally divided between the jumps marked *K*_4_ and *K*_5_. See Figure 6(b) for the corresponding weights in our simple helical example (the weights on unmarked edges are 1). This mapping is specified in the symmetry definition file; see Supporting Material S1 for an example.

#### Minimizing asymmetric whole-structure energies

Another difficult case that arises comes about when a whole-structure energy is applied asymmetrically, that is, the energies for each subunit are not equal. This commonly arises with experimental electron density data, if not symmetrically averaged, but may also arise with coordinate constraints or other types of experimental data, where gradients are *not* identical (save for a symmetric transformation) in each subunit.

Unfortunately, this case is not directly handled by Rosetta's symmetry machinery, as the symmetric modeling is built on the idea that each energy term only differs by a symmetric transformation between subunits. However, one may get around this limitation by making the score function “symmetry-aware”. The basic idea is to map all the derivatives to the master subunit. For every atom in the symmetric complex, the gradient is computed. Then, for each atom in each slave subunit, the symmetric rotation mapping the subunit to the master subunit is applied to the gradient. These are then added to the corresponding atom in the master subunit's gradient.

**Figure 7:**
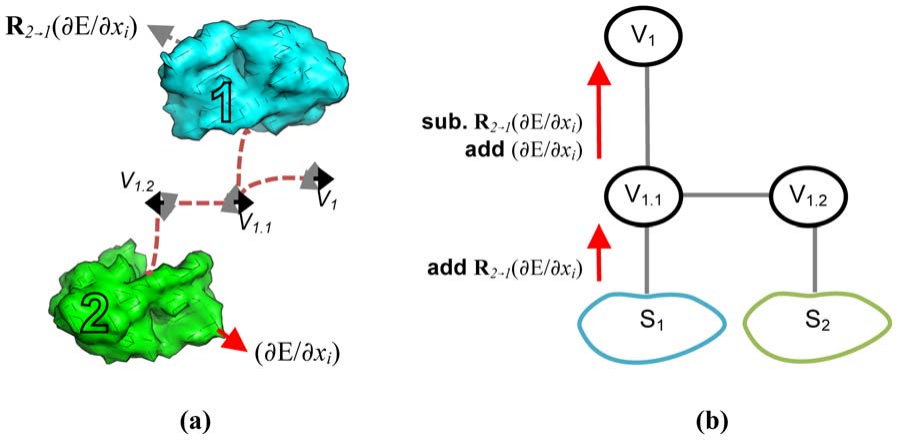
An illustration of a problem that arises when whole-structure energies are minimized. **(a)** A homodimeric system in Rosetta can allow movement of the rigid-body orientation between subunits (the jumps between *V_1.X_* and the subunits) as well as the rigid-body orientation of the entire system (the jump between *V_1_* and *V_1.1_*; *V_1.1_* and *V_1.2_* are connected with a “fixed jump” that maintains the symmetry of the system). Contributions to the energy gradient from atoms in slave subunits are treated differently in these two jumps. When minimizing the orientation between subunits, the gradient (∂E/∂*x_i_*) with respect to some atom *x_i_* in a slave subunit should be rotated to the master subunit, using the transformation **R** *_2→1_*. When minimizing this minimizing the orientation of the whole system, this gradient should not be rotated. **(b)** This can be handled in Rosetta by applying “correction factors” to the gradients of the virtual residues. To illustrate, when computing the gradients of atoms of S_1_, we add the rotated gradients of S_2_. Then, at virtual V_1.1_, we subtract the rotated gradients, and add the unrotated gradients.

When the symmetry group is a single layer hierarchy, and the rigid-body orientation of the whole system is not allowed to move, this works as expected. However, when the symmetry hierarchy is multi-layer, or the whole system is allowed to move as a rigid body, then there are problems minimizing along jumps within the symmetry hierarchy. It is clear to see this when we consider the C_2_ symmetry shown in Figure 7(a). In this case, the rigid-body configuration of the entire system is allowed to move. Suppose the gradient of some atom in subunit *2* points “down”. As we map this to *1*, we rotate so that the gradient points up. Indeed, when minimizing along the jump between *1* and *2*, this behavior is correct: the downward gradient pulls *1* and *2* apart, that is, *2* downward, and *1* upward. However, this same gradient has the opposite effect when we consider the rigid-body orientation of the entire system: the downward gradient in *2* should not be rotated when mapped to *1*.

This may be done within Rosetta by storing the unrotated derivatives for every atom in the symmetric complex. With backbone and sidechain torsions, the naive strategy – rotating each subunit to the master one – may be used. Then, at each symmetric jump, the transformation mapping the parent virtual residue to the master's virtual residue at the same level in the hierarchy is applied. Since the lower levels in the hierarchy have already added their layer's rotated gradients, this can be handled by assigning a “correction gradient” to the virtual residues within the upper levels of the symmetry hierarchy. That is, the virtual residue is assigned a “gradient” that is the result from subtracting all the previously rotated gradients and adding the all the newly rotated gradients of every atom in the subtree beneath it. Figure 7(b) illustrates this idea graphically.

Finally, notice that we use the term gradient loosely here. Rosetta's implementation uses the recurrence of Abe et al. to pass along two components of the gradient up the fold tree, denoted *f*_1_ and *f*_2_, which allows for efficient conversion between Cartesian space and torsion space gradients. In this case, the correction factors associated with virtual residues are applied directly to these *f*_1_s and *f*_2_s, instead of the gradients.

Within Rosetta3, this is currently only implemented for the symmetry-aware electron density scoring function. The code for this may be found in src/core/scoring/electron_density/ElectronDensityEnergy.cc.

### Applications of Symmetric Modeling

In this section, we provide a practical guide to modeling symmetrical structures with Rosetta. We first describe the file format by which symmetry information is encoded. Then, we introduce four different symmetry-enabled Rosetta applications (symmetric docking, fold-and-dock, comparative modeling and fixed backbone design) and describe how they may be configured to make use of the symmetry machinery. Together with this manuscript we distribute a set of canonical test cases as Supporting File S1 for these four applications.

### Symmetry definition files

Everything that Rosetta needs to know about the symmetry of the system is encoded in the symmetry definition file (SDF), which is provided as input to any Rosetta protocol run with symmetry. This file provides: *(i)* information about how to generate the rigid body symmetry of the molecular system, *(ii)* how to calculate the total energy of the systems from a subset of modeled subunits, *(iii)* how to calculate energy gradients, *(iv)* what the rigid body degrees of freedom are, *(v)* how to generate the initial configuration of the symmetric system, and *(vi)* how the system may be perturbed while maintaining symmetry. Given the complexity of the required information, we provide two scripts to generate SDFs for the most common symmetries and modeling tasks, which are described in the next section. However, sometimes a problem requires customization or even “handcrafting” of SDFs. In this section we provide a brief description of the key elements of the syntax of SDFs. A full review can be found in Supporting Material S1.

#### Overview of the format

**Figure 8:**
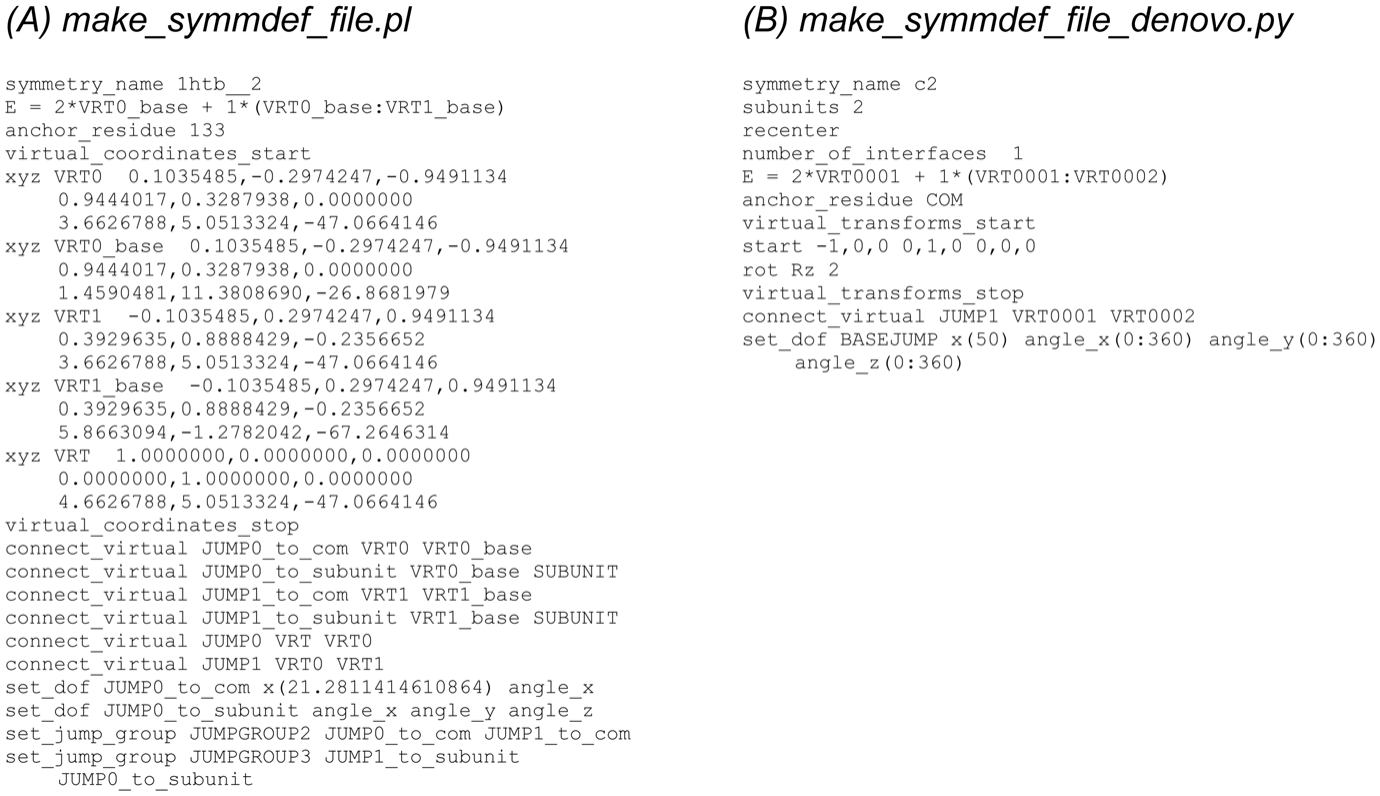
Examples of two symmetry definition files for C_2_ symmetric systems. **(a)** Symmetry definition file for alcohol dehydrogenase (pdb id 1htb) generated by *make_symmdef_file.pl*. **(b)** Symmetry definition file generated by *make_symmdef_file_denovo.py* for denovo structure prediction.

Figure 8 shows two SDFs for setting up the C2 symmetry. One of them is generated by application of the *make_symmdef_file.pl* script to the dimeric crystal structure of alcohol dehydrogenase, while the other is generated by make*_symmdef_file_denovo.py* without a structure input. These scripts each serve a different purpose: *make_symmdef_file.pl* is used to create symmetry information by replicating the symmetric transformation (including the global coordinate frame) from a preexisting symmetric complex, which may be used in docking perturbation studies or comparative modeling; while *make_symmdef_file_denovo.py* is used to “bootstrap” symmetry information in the absence of a preexisting structure, as in *de novo* modeling.

One of the key purposes of the SDF is to inform Rosetta how to evaluate the energy of a structure in a symmetric fashion. In the SDF for alcohol dehydroganase in Figure 8 the following line provides a recipe to calculate the energy of the dimer by evaluating only a subset of the atomic interactions:

E  =  2*VRT0_base + 1*(VRT0_base:VRT1_base)

In this example, the subunit that is connected to the virtual residue *VRT0_base* is the scoring subunit and the internal energies in this subunit is multiplied by a factor of 2 to get the total system energy. Then intermolecular energies from the subunit connected to *VRT1_base* is added with a factor of 1.

A second key aspect of the SDF is to provide the coordinates of the reference frames – that is, the virtual residues – to set up the rigid body symmetry. There are two ways of specifying these coordinate frames: *explicitly*, through specification of their coordinates in Cartesian space together with the unit vectors specifying the X and Y axis of the reference frame, or *implicitly*, by application of a series of rotation and translation operations on a single virtual residue. In the alcohol dehydrogenase SDF the virtual residues are explicitly specified:

virtual_coordinates_start

xyz VRT0 0.1035485,-0.2974247,-0.9491134 0.9444017,0.3287938,0.0000000 3.6626788,5.0513324,-47.0664146

…

virtual_coordinates_stop

Here the virtual residue named *VRT0* is specified by three triplets encoding *x* and *y* unit vectors together with the origin. Alternatively, these virtual coordinates can be encoded implicitly, by application of rotation and translation operations. In the second SDF in Figure 8, the virtual coordinates are specified as:

virtual_transforms_start

start -1,0,0 0,1,0 0,0,0

rot Rz 2

virtual_transforms_stop

This specifies that the first virtual residue is encoded by the triplets defined after the start keyword. A second virtual residue is generated by application of twofold rotation around the Cartesian Z axis (rot Rz 2) on the start virtual residue.

A third key aspect of the SDF is specifying what dofs in the system are allowed to move, what their initial values should be and how to perturb them. In the SDF for alcohol dehydrogenase, the line:

set_dof JUMP0_to_com x(21.28) angle_x

specifies that for the jump named *JUMP0_to_com*, translation along *x* and rotation around the *x*-axis are allowed. The placement along *x* is also initialized (to a value of 21.28).

### Scripts for making symmetry definitions

Generally, symmetry definition files will not be hand-crafted, but rather, will be created by a script. There are two such scripts included with Rosetta3. The first of these, *make_symmdef_file.pl*, creates an SDF that recapitulates the symmetry from a PDB file. The second of these, *make_symmdef_file_denovo.py*, makes SDFs in a canonical coordinate frame, for use in symmetric docking or *de novo* folding with fold-and-dock.

#### make_symmdef_file.pl

This script automatically creates symmetry definition files corresponding to the symmetry in some template protein structure. If the template is not symmetrical – for example, if differing crystal contacts between subunits cause some asymmetry – then it is “symmetrized” by the script. For these cases, simple heuristics are used to find a symmetric system nearby the target system. However, if the starting model is very asymmetric, the symmetrized structure may be very far from the input. Generally this is undesirable, and suggests modeling the system asymmetrically.

The script provides at least limited support for most types of point, helical, and lattice symmetries. However, there are some caveats. The following symmetry types are currently unsupported by the script:

- Tetrahedral, octahedral and icosahedral point symmetries are improperly generated.
- Nonpolar helical symmetries (a D*_n_* point group at each helical translation) are not understood by the script. Apolar helical symmetries (a C*_n_* point group at each helical translation) are handled properly.
- 2D lattice (or wallpaper) symmetry is not created by the script.
- 3D lattice (or crystal) symmetries are available, but assume a fixed unit cell size. Systems produced in this manner allow rigid-body movement of a subunit in the asymmetric unit, but do not allow the cell dimensions to change during simulation.

The script runs in one of three modes, depending on the symmetry type: noncrystallographic (point) symmetries, crystallographic symmetry, and helical symmetry. The mode of the script is specified with the flag

-m {NCS|CRYST|HELIX}

If this flag is not given, *-m NCS* is assumed.

There are several options common to each mode:

-p <string>

input PDB file

-r <real>

the max Cα-Cα distance between two interacting chains

When the system is constructed, a master chain is first selected (how this is specified is mode-specific). The resulting SDF specifies a system where the only subunits that are explicitly modeled are those with some Cα within the specified interaction distance of the master subunit.

For noncrystallographic symmetry mode (*-m NCS*), several other options are used to specify the master chains, and the point symmetry groups to expand.

-a <char>

the chain ID of the main chain

-i <char>

the chain IDs of one chain in each symmetric subcomplex

Use of the -*a* option is straightforward, however, the -*i* option may be a bit tricky. With the -*i* flag, a single adjacent neighbor in each point group must be given, regardless of the number of subunits in the point group: one would specify ‘*-a A -i B*’ for both C_2_ and C_38_ symmetry (assuming A and B were adjacent chains). To generate the SDF for C_2_ symmetry in Figure 8(a) the following command can be be employed:

make_symmdef_file.pl -m NCS -p input.pdb -a A -i B > C2.symm

As another example, with a D4 symmetric system, with chains A-B-C-D in the upper ring and chains E-F-G-H in the lower ring, one would specify

make_symmdef_file.pl -m NCS -p D4.pdb -a A -i B E -r 12 > D4.symm

Alternately, one could specify the interacting chains in reverse order, as ‘*-i E B*’. This would create a different hierarchical representation of the same symmetry, and if the input PDB had some asymmetry, then the corresponding symmetrization would be slightly different. If the input system is not perfectly symmetric, it may be worth trying different chain combinations to minimize the residual error of symmetrizing the system. Finally, if the SDF wants to allow movement of the rigid-body orientation of the entire system, then an additional flag may be used:

-e

allow rigid body minimization of complete system

This flag is important when the structure is scored against experimental data that depends on the rotation or the whole system, such as electron-density or RDC data.

For crystallographic symmetry mode (*-m CRYST*), the inputs are a bit simpler. The ‘*CRYST1*’ line in the input PDB file is used to define the symmetric system. Alternately, one may provide values on the command line instead:

-c <real>x6

override the unit cell parameters in the PDB file with these values

-s <string>

override the spacegroup in the PDB file with these values

The resulting SDF defines a system where a single subunit is placed in its “lattice context,” where only the symmetric copies that interact with the master subunit are explicitly represented. As a sidenote, the energy line in the SDF specifies the energy calculated by Rosetta as twice the per-subunit energy.

Finally, helical symmetry mode (*-m HELIX*) provides options for specifying the master chain, an adjacent lattice chain, and a point-group chain:

-a <char>

the chain ID of the main chain

-b <char>

the chain ID of the next chain along the fiber/helix

-i <char>

the chain ID of a chain in -a's point symmetry group

A helical twist can be forced by appending:*n* to the helical chain. For example, the following command script forces a helix with 3 subunits per turn:

make_symmdef_file.pl -m HELIX -p fiber2.pdb -a A -b B:3 > fiber3.symm

The same heuristics used to symmetrize a system are used to force a different helical twist. Thus, if this value is very different from the twist provided in the PDB, then the system may move dramatically.

When run, the SDF that recapitulates the symmetry in the input PDB is written to stdout. Several PDB files are written as well. Given the input file *input.pdb*, the script will write out up to three files:

*input*_symm.pdb

the symmetrized version of the input file, showing the complete point symmetry group.

*input*_model_AB.pdb

the same as above, but only showing chains that form an interface with chain A

*input*_INPUT.pdb

the input PDB to Rosetta's symmetry modeling, the coordinates of the master subunit (typically a single chain in the symmetric complex).

The files *input_symm.pdb* and *input_model_AB.pdb* are provided for two purposes. First, they are a check to verify that the symmetrization heuristic did not move the system too far from its start point. Second, they show the difference between the complete symmetric system, and the parts of the system Rosetta is explicitly modeling. For mode CRYST, only the *input_symm.pdb* file is created, as the input PDB is expected to contain only the asymmetric unit. When running Rosetta with symmetry, the input structure passed to Rosetta is the monomer model *input_INPUT.pdb* (or *input.pdb* in the case of CRYST); the *symm* file written to stdout is also given as an input with the flag -symmetry_definition.

#### make_symmdef_file_denovo.py

When the structures of symmetric protein assemblies are predicted *de novo* the starting point is either a protein sequence or the structure of a single subunit. Therefor the *make_symmdef_file.pl* script is not a straighforward tool for generating a SDF. In principle, a protein complex with identical symmetry can be used as the starting point to generate a SDF using the *make_symmdef_file.pl* script. The resulting SDF has to be modified by hand to remove any dependence on the rigid body position of the analyzed complex and to completely randomize the symmetric rigid body space. Alternatively, the *make_symmdef_file_denovo.py* script can be used to generate a SDF. This script takes as an input the symmetry type being simulated, the number of subunits in the full system and a specification of whether only a subsystem of the system should be simulated. Currently the script is limited to C*_n_* and D*_n_* symmetries. To generate the SDF for C_2_ symmetry in Figure 8(b) the following command can be employed:

make_symmdef_file_denovo.py -symm_type cn -nsub 2 > C2.symm

A SDF for D_2_ symmetry can be generated with:

make_symmdef_file_denovo.py -symm_type dn -nsub 4 > D2.symm

By default the script encodes for all subunits to be simulated. For larger complexes, such as a 38-membered ring, a subunit only interacts with its direct neighbors in the ring and its not necessary to simulate all subunits (see Figure 4). For really large systems neighbor detection, kinematics and system memory will become major bottlenecks. Thus, it is suggested to limit the simulated system to a smaller subsystem in these cases. This can be achieved by adding the flag *-subsystem* to the command line:

make_symmdef_file_denovo.py -symm_type cn -nsub 38 –subsystem > C24.symm

This generates a SDF that encodes only 3 out of 38 subunits.

Symmetry options that control protocol behavior can also be defined in the SDF. *De novo* prediction of protein complexes typically involves translational moves to bring subunits into contact. For systems with multiple translational dofs there are several ways to select the order of the translational moves. These preferences can be specified by an additional set of flags (*-slide_type*, *-slide_criteria_type* and *-slide_criteria_val* flags). The meaning of these flags is described in greater detail in Supporting File S1.

To generate SDFs for symmetries outside C*_n_* and D*_n_* there are two alternatives. First, SDFs can be written by hand. Second, a protein complex with identical symmetry type can be used as a starting point. As mentioned, that will require manual editing of the SDF.

#### Making a Rosetta protocol aware of symmetry

The symmetry machinery in Rosetta3 is built to take advantage of the object-oriented architecture of Rosetta's core. Polymorphism and inheritance allows symmetric versions of key components in Rosetta's scoring, kinematics, sidechain-optimization, and minimization machinery to be plugged in in place of their non-symmetric counterparts, which allows symmetry to be used with minimal adjustment to the code. When adapting a scientific protocol to use symmetry, care must be taken that the symmetric versions of these classes are employed. For most protocols, if Rosetta is given a symmetry definition file, this is automatic, and Rosetta will protect you from making the system nonsymmetric, but care must be taken in protocols where kinematic connectivity or the coordinates of the protein change, to make sure that the symmetric complex is perturbed in a reasonable manner. Typically, changes to the conformation of a protein are controlled through higher-level objects called movers that interface with the lower level core functions. There are symmetrical versions of the most common movers, which substantially simplify the adaptation process.

First, instantiation of symmetry at the beginning of a protocol involves a check for the presence of a symmetry definition file specified on the command line followed by a call to a mover that initialize the symmetry information by reading from a SDF and swapping in symmetrical versions of base classes for energy evaluation and coordinate storage into the Pose object (the Pose object represents the complete state of the molecular system).

if (option[OptionKeys::symmetry::symmetry_definition].user()) {

moves::MoverOP setup(new moves::symmetry::SetupForSymmetryMover);

setup->apply(pose);

}

A number of utility classes are available to get access to symmetry information and to make objects compatible with symmetry, including core/pose/symmetry/util.cc and core/conformation/symmetry/util.cc. A typical step in adapting a protocol for symmetry is to check for the presence of a symmetrical Pose object and control the instantiation of a mover based on the result of the query:

moves::MinMoverOP min_mover;

if (pose::symmetry::is_symmetric(pose)) {

min_mover  =  new moves::symmetry::SymMinMover(...);

} else {

min_mover  =  new moves::MinMover(...);

}

min_mover->apply(pose);

Directly setting torsions or jumps in the master subunit (or using nonsymmetric movers that only do this) is fine: the symmetric machinery will maintain the symmetry of the overall system. For a great many protocols, the only changes necessary to enable symmetry are the two shown above.

## Results and Discussion

Finally, a number of protocols have been ported to use symmetry if a symmetry definition file is provided. In addition to Rosetta's *relax* protocol – for all-atom refinement of symmetric systems – four commonly used protocols have been heavily tested for use with symmetric complex modeling.

### Symmetric docking

The symmetric assembly protocol aims to predict the structure of a symmetrical protein assembly based on the structure of a single subunit [1]. It has been shown to accurately predict the structure of protein assemblies with cyclical, helical and icosahedral symmetries. In the start of each simulation all simulated subunits are placed in a configuration that avoids atomic contacts between subunits and adopts the overall symmetry of the system. Random starting points are generated by randomization of rotational degrees of freedom. The simulation proceeds by translation of the subunits along all their symmetrically allowed dofs in order to establish atomic contact between subunits (called slide moves). Depending on the type of symmetry, this may involve sliding along several directions. For C*_n_* symmetry there is only one translational dof and hence one slide direction. For D*_n_* symmetry there are two translational dofs and in order to sample the whole symmetric configurational space sliding in two directions is required. The slide procedure for multidimensional slides can be customized as described in the description of the symmetry format in Supporting Material S1.

When atomic contacts have been established the protein complex energy is optimized in a rigid body Monte Carlo search performed using a low-resolution knowledge-based scoring function and a simplified representation of the protein. All dofs described in the SDF are perturbed during the Monte Carlo procedure. The low-resolution phase is followed by further refinement in Rosetta's high-resolution energy function, with an all-atom representation of the protein assembly. The energy is optimized using a Monte Carlo minimization procedure, which consists of several of cycles of rigid body moves followed by symmetric side-chain optimization and symmetric energy minimization.

To run the symmetric docking protocol, two pieces of input data are required: the structure of a protein subunit and a SDF. A preexisting symmetric protein complex can be refined using the docking protocol (for perturbation studies, for example), with the starting input subunit and SDF generated by the *make_symmdef_file.pl*. For *de novo* structure prediction the script *make_symmdef_file_denovo.py* is used instead. For the most common symmetry type, C2, the following command can be employed to generate the SDF:

make_symmdef_file_denovo.py -symm_type cn -nsub 2 > C2.symm

The reference frames encoded by this script have their axis pointing towards the absolute origin (0,0,0) in Cartesian space and with the translational dof along the Cartesian x-axis. Thus, it is important that the axis connecting the anchor residues align with the Cartesian *x*-axis so that translation along Cartesian *x* leads to atomic contact between subunits. The recenter item in the SDF ensures this.

The default *set_dof* line in the SDF:

set_dof BASEJUMP x(50) angle_x(0:360) angle_y(0:360) angle_z(0:360)

This line specifies that the two subunits will initially be placed at (50,0,0) and (-50,0,0). The 100 Å distance is typically large enough that the subunits start in a non-contacting configuration. The initial positioning can be changed by manually editing this line of the SDF.

The last three terms in this line describe the orientation parameters of the jump:

angle_x(0:360) angle_y(0:360) angle_z(0:360)

In this particular case, the rotational dofs should be completely randomized. Normally, when a range is given a random value is found in the range and a rotation of that angle is applied by rotation around the given axis. However, when all three rotations are given the range 0-360 degree, Rosetta ensures that rotational space is uniformly sampled.

As well, the presence of multiple identical subunits presents some problems when calculating root-mean-square (rms) deviation values to a reference structure. For complexes with more than two subunits, it may be necessary to consider alternate chain orderings in order to find the lowest rms value. A symmetric rms value can be determined with Rosetta by addition of a command line flag (-symmetry:symmetric_rmsd).

A typical prediction case for symmetric docking is distributed in Supporting File S1. A brief discussion of options is included.

### Fold-and-dock

The fold-and-dock protocol simultaneously samples the internal degrees of freedom of a monomer, and rigid-body degrees of freedom between (symmetrically disposed) monomers. It is well suited to predicting the structures of intertwined symmetric assemblies for which the structure of the monomer is not stable in isolation, and hence not amenable to a two stage approach in which monomer predicted structures are first generated in isolation and then docked together [10]. The fold-and-dock protocol has been used to accurately predict complexes with C*_n_* and D*_n_* symmetry for proteins with subunit sizes up to 100 residues [10]. This size range can be extended for systems with experimental data from NMR spectroscopy.

Fold-and-dock is a combination of the symmetric assembly protocol and the Rosetta abinitio structure prediction protocol. Like symmetric docking, the simulation starts with a randomized symmetric configuration of subunits with no atomic contacts between subunits. The protein subunits initially adopt an extended structure. What follows is a simulated annealing fragment assembly – of the same kind used for regular monomeric abinitio structure prediction – performed symmetrically. In addition, two types of rigid body moves are attempted at random frequency: a *slide move*, to translate subunits into atomic contact, and random perturbation of rigid body dofs specified in the SDF. Fragment assembly is performed using a simplified representation of the protein and with a low resolution scoring function. This initial process is followed by all-atom refinement in Rosetta's high-resolution energy function [10]. Fold-and-dock requires three types of data input: one subunit's sequence, a simple configuration file (topology broker file) and an SDF. The SDF is generated as described in the symmetric docking section.

The models produced by *de novo* structure prediction are typically analyzed with similarity clustering. The clustering application can be modified to calculate a symmetry-aware rms as described in the symmetric docking section.

A typical prediction case for fold-and-dock is distributed in Supporting File S1. A brief discussion of options is included.

### Symmetric comparative modeling

Another case where symmetric modeling is beneficial arises with comparative (or template-based) modeling of symmetric structures. When building a homology model of a symmetric multimer, if a template contains the same symmetry, it may be reasonable to use the symmetry of the template when building the threaded model of the target.

Rosetta's comparative modeling protocol performs threading of the target sequence onto the template backbone, followed by fragment-based rebuilding of gaps in the threaded model, and finally all-atom optimization of Rosetta's energy function. By running the symmetry definition script *make_symmdef_file.pl* on the template structure to create an SDF, and giving that symmetry definition file (along with a monomer of the template) to Rosetta's comparative modeling protocol, both fragment-based gap rebuilding and all-atom optimization take into account the symmetric context of the model. This protocol was used on multiple targets in the most recent CASP9 [11] experiment.

A typical prediction case for comparative modeling is distributed in Supporting File S1.

### Symmetric design

The fixed backbone design provides a direct interface to Rosetta's sidechain optimization module, the packer. Through the design application the energy of a protein subunit or complex can be minimized by optimization of the protein sequence. The symmetrical version of the packer is invoked by specification of a SDF file on the command line. The *make_symmdef_file.pl* script is typically used to generate a SDF and the monomeric input structure. Configuration makes use of *resfiles*, which provides residue-level control of the packer. For symmetric systems, these are only specified for one subunit.

A typical prediction case for fixed backbone design is distributed in Supporting File S1.

### Comparison with Rosetta2

We have previously modeled symmetrical protein assemblies using an implementation in Rosetta2, described in [1]. The symmetry machinery was used to develop methods for symmetric docking [1] and simultaneously folding and docking [10]. In both of these works the result of prediction benchmarks were presented. To confirm that the new implementation in Rosetta3 performs as well as Rosetta2 in scientific terms we have repeated predictions for a majority of the proteins in these two benchmark sets and shown that similar quality of predictions is reached (data not shown). In addition, we have recently shown that the structure of large homomeric protein complexes can be determined by combining limited NMR data and symmetric modeling (ab initio structure prediction followed by symmetric docking, or fold-and-dock) [12], which establishes that symmetrical modeling in Rosetta3 can be used to generate high-quality structural models.

The object-oriented nature of Rosetta3 enables us to take full benefit of structural symmetry in protein modeling. Rosetta3 can, given the right symmetry definition, model all types of symmetries. In contrast, Rosetta2 only included a few hard-coded symmetry types and exact values of energy gradients for more complex symmetries (such as helix symmetry) could not calculated, as it required of the types of corrections described in the energy minimization section. The inflexible nature of Rosetta2 code base prevented the implementation of several features present in Rosetta3. A major benefit of Rosetta3 over Rosetta2 is that lists of atom and residue neighbors, used during energy calculation and energy minimization, is restricted to only those pairs that are required to evaluate the energy of the whole system. With Rosetta2 the memory requirements gets prohibitory large for big systems and a substantial fraction of the running time is spent on generating updated neighbor list.

We have compared the running time of Rosetta3 with Rosetta2 for several prediction protocols and molecular systems. Such comparisons put the improvements in practical terms but it cannot be used to isolate the effects of modifications to the symmetry machinery. This is because general improvements of Rosetta3 from Rosetta2, together with protocol level developments, will also impact running times. The comparison here is with standard parameters in Rosetta2 and 3. A symmetric docking run on a C_2_ system with 164 residues is 2.3 times faster in Rosetta3 than Rosetta2. For more computational intense protocols and with more complex symmetries the improvement is larger. Running fold-and-dock on a sequence with 100 residues with D_2_ symmetry (4 subunits with in total 400 residues) the run time of Rosetta3 is around 13 times shorter per model than Rosetta2. For a dimer with the same number of residues with C_2_ symmetry the improvement is 26 times. Rosetta2 scales poorly with the number of simulated subunits in the system (runs with several hundred residues is unpractical both due to memory and speed issues) while Rosetta3 can model quite large protein assemblies without a dramatic increase running time. We have determined running time of the fold-and-dock protocol for a system with 100 residues with C*_n_* symmetric systems ranging from dimers up to 10mers. The running time increases roughly linearly with 70s per added subunit and model. When going from a dimer (with 200 residues) to a 10mer (with 1000 residues) the running time increases around 5 times. For very large systems the energy calculation and minimization no longer is the bottleneck. Instead updating the three-dimensional coordinates of all modeled atoms take up a large fraction of the running time.

The run time of a symmetric modeling run depends on the size of the system (mostly the subunits size), the number of degrees-of-freedom in the rigid body sampling and the scientific protocol. As a reference, generating all-atom model of a D_2_ symmetric 100-residue protein using fold-and-dock takes about 8 minutes on contemporary desktop computer.

### Conclusion

We present a general framework for the modeling and design of symmetric protein assemblies. The current implementation presents a set of ready-made scientific protocols to facilitate some common tasks in structural biology: structure prediction of symmetric protein structure from sequence or subunit structure, comparative modeling of symmetric proteins or proteins in crystal lattices and symmetric protein design. The list of symmetry-enabled protocols can easily be extended by small modifications to the Rosetta source code. In the same manner, the framework can be used to model more exotic symmetry types, not currently covered by the distributed scripts, without any changes to the source code. Thus, this manuscript describes the extension of the Rosetta methodology to the exciting universe of symmetric protein assemblies.

## Supporting Information

Material S1 A complete reference guide to Rosetta3 symmetry definition files.
(DOC)

File S1 An archive containing example flags file and input files for running four different symmetry protocols in Rosetta3. The protocols include symmetric docking, symmetric comparative modeling, fold-and-dock, and symmetric design.
(BZ2)
