# Supplementary material for: Modeling Symmetric Macromolecular Structures in Rosetta3
Source: PLoS One. 2011 Jun 22;6(6):e20450. doi: 10.1371/journal.pone.0020450 (PMC3120754; doi:10.1371/journal.pone.0020450)
Supplement: Material S1 — A complete reference guide to Rosetta3 symmetry definition files. (DOC) [file pone.0020450.s001.doc]

### **Supporting Material**

Below we give a detailed reference of the syntax of the symmetry definition files, using the definitions in Figure 8 as a point of reference:

symmetry_name 1htb__2

A string describing the symmetry of the system. This may be anything.

E = 2*VRT0_base + 1*(VRT0_base:VRT1_base)

This informs Rosetta how to symmetrically score the structure. In this example, the subunit that is connected to the virtual residue VRT0_base is the scoring subunit and the internal energies in this subunit is multiplied by a factor of 2 to get the total system energy. Then intermolecular energies from the subunit connected to VRT1_base are added with a factor of 1.

anchor_residue 133

Each subunit is anchored by jump to its residue 133 (using a numbering starting from 1 to the number of residues per subunit).

virtual_coordinates_start

xyz VRT0 0.1035485,-0.2974247,-0.9491134 0.9444017,0.3287938,0.0000000 3.6626788,5.0513324,-47.0664146

...

virtual_coordinates_stop

This section defines all the reference frames of the system by specifying the virtual residues.

There are three triplets per virtual residue (X, Y and ORIGIN) that each have three coordinates describing units vectors (for X and Y) and a center (ORIGIN). For example, VRT reference frame is defined by an X-axis pointing in the direction of the Cartesian *x* (1,0,0), a *y*-axis pointing in the direction of the Cartesian *y* (0,1,0) and is placed at the absolute coordinates (4.6626788, 5.0513324, -47.0664146). The names of the virtual residues may be chosen freely.

connect_virtual JUMP0_to_com VRT0 VRT0_base

...

A set of *connect_virtual* statements encode all the jumps in the system. For example, consider the line:

connect_virtual JUMP0_to_com VRT0 VRT0_base

This line indicates that virtuals *VRT0* and *VRT0_base* should be connected by a jump. We name this jump *JUMP0_to_com*. Any character string can be chosen for this name. If the second virtual is “*SUBUNIT*” then it means that a jump from a virtual to a subunit is specified (connected to anchor residue, 133 in this case).

set_dof JUMP0_to_com x(21.2811414610864) angle_x

set_dof JUMP0_to_subunit angle_x angle_y angle_z

A set of *set_dof* statements specify which degrees of freedom are allowed for a particular jump. They are *x*,*y*,*z* for translations and *angle_x*, *angle_y*, *angle_z* for rotations. In this particular case, translations along the *x* (but not *y* and *z*) axis and rotation around *x* (but not *y* and *z*) for the jump defined by *JUMP0_to_com* are allowed; rotations around *x*, *y*, and *z* (but no translations) for the jump defined by *JUMP0_to_subunit* are allowed. These dofs are inherent to the particular symmetry of the system. The *set_dof* should only be set for the master jump. If a jump does not have a *set_dof* statement associated with it then by default the jump is unmovable. The jump is initialized along the *x* direction with a value of 21.2811414610864. The initial placement of subunits can be encoded by modifying this line. For example, consider the following *set_dof* statement:

set_dof JUMP0_to_com x(10:20) angle_x

This specifies that *x* should be randomly chosen in the range 10-20. For angles:

set_dof JUMP0_to_subunit angle_x(360) angle_y(360) angle_z(360)

This tells Rosetta that the rotational dofs should be completely randomized for this jump. Rigid body perturbation sizes can also be encoded by adding a second range:

set_dof JUMP0_to_com x(10:20;3) angle_x c2n

In this case the perturbation size for the *x* translation dof is chosen to be 3. Finally, the direction of a jump can be controlled. The default is *n2c* while *c2n* reverses the direction of the jump. It is up to the protocols to honor these jump parameters (range and directions). That can be achieved by calling special (symmetry adapted) versions of functions that are responsible for changing rigid body positions.

set_jump_group JUMPGROUP2 JUMP0_to_com JUMP1_to_com

set_jump_group JUMPGROUP3 JUMP1_to_subunit JUMP0_to_subunit

A set of *set_jump_group* statements tells Rosetta the jumps that are “cloned” in master/slave relationships. The first jump in the jump group is the master. The name of the jump group can be chosen freely. When minimizing with lattice symmetries, as described in the minimization section, energy gradients calculated across certain jumps may need to be reweighted in order to account for the domino effect generated by jumps coupled to jumps. This can be done while specifying the jump groups. *JUMP_0_2:3* specifies that energy gradients across *JUMP_0_2* should be divided by a factor of 3.

In many modeling applications, such as *de novo* structure prediction, the initial placement of the subunits must be randomized while maintaining the overall symmetry. Here, coordinates of an initial symmetric complex from which the placement of virtual residues can be constructed are missing. As an alternative to the *virtual_coordinates_start* / *virtual_coordinates_stop* setup, the reference frames can be generated by application elemental rotation/translation operations. Figure 8(b) shows a simplified SDF file suitable for denovo modeling of a C2 complex. This provides a simplified method to generate new SDFs based on geometrical reasoning. The virtual residues are defined by *virtual_transforms_start* / *virtual_transforms_stop* section:

virtual_transforms_start

start -1,0,0 0,1,0 0,0,0

rot Rz 2

virtual_transforms_stop

This section places virtual residues by application of a rotation operation *rot Rz 2*, which specifies a twofold rotation around the Cartesian *z*-axis. An initial virtual residue is placed by the statement *start -1,0,0 0,1,0 0,0,0* which encodes a (X, Y, ORIGIN) triplet. The first transform will be applied to this virtual to generate the second. Rotations can be specified in degrees as well: e.g., *rot Rz_angle 180*. The statement *rot Rz 2* encodes two *Rz_angle 180* rotations. Each rotation operation generates a new virtual residue; subsequent virtuals are constructed by applying the next rotation operation to this virtual. In aggregation, this generates virtual residues at:

$$\mathbf{R}_{z}^{i}(180^{\circ})\cdot start,\text{ }i=1,2$$

Translations can also be specified: *trans 4,5,2* encodes the translation vector (4,5,2).

virtual_transforms_start consecutive

The consecutive keywords signals that for every virtual that is placed all the transformations between *virtual_transforms_start* and *virtual_transforms_stop* will be applied before generating the coordinates.

When specifying the virtual residues using the virtual transforms the virtual residues are automatically named *VRT0001*, *VRT0002* etc. Jumps to subunits are automatically generated and a jump group is generated for the subunit jumps. Jump groups may be overridden using a *set_jump_group* statement.

recenter

Tells Rosetta to recenter the input subunit so that the CA atom of the anchor residue is at the origin (0,0,0). This ensures that a subunit is placed on the axis symmetry axis, something that is of importance when using the simplified SDFs in denovo modeling.

anchor_residue (com|COM)

Instead of specifying a numeric value for the anchor residue it can be selected as the residue closest to the center-of-mass of the subunit by using the keyword *com* or *COM*.

In addition to these generic options there are other keywords that can be used to specify the behavior of symmetry related features in scientific protocols. One such feature is control of translation of subunits along directions that maintain the symmetry of the system. In denovo modeling subunits are translated along all symmetric dofs to establish atomic contact between the subunits. These type of rigid body moves are called slide moves. For more complicated symmetries translations along several dofs may be necessary to establish contact. The order in which contact is established by sliding moves along the symmetric dofs is controlled by statements in the SDF:

slide_type (RANDOM|SEQUENTIAL|ORDERED_SEQUENTIAL)

Controls how multidimensional slide-into-contact moves are made. For example, for Dn symmetry there are two sliding directions. A slide can be done by randomly selecting a slide direction for each slide step (*RANDOM*), randomly deciding on which direction should be slided first but always sequentially go through both (*SEQUENTIAL*), or define the order yourself (*ORDERED_SEQUENTIAL*). *RANDOM* is the default option.

slide_criteria_type (CEN_DOCK_SCORE|FA_REP_SCORE|CONTACTS)

Defines what the criteria is for abandoning a slide move. Either the *CEN_DOCK_SCORE*, *FA_REP_SCORE* or the number of contacts is used as the criteria. *CEN_DOCK_SCORE* is the default criteria.

slide_criteria_val (value|AUTOMATIC)

Sets the actual value when a slide move is abandoned given the criteria type. By default set to *AUTOMATIC*, which means that Rosetta figures it out by itself. Setting a numeric value is typically only useful for the *CONTACTS* slide type.

slide_order jump name

If the *ORDERED_SEQUENTIAL* slide type is used the order by which the jumps corresponding to the allowed translations are visited can be specified. Uses the jump names defined in the SDF.
